# Attention Capture of Non-target Emotional Faces: An Evidence From Reward Learning

**DOI:** 10.3389/fpsyg.2019.03004

**Published:** 2020-01-31

**Authors:** Xing Zhou, Bixuan Du, Zhiqing Wei, Weiqi He

**Affiliations:** ^1^Research Center of Brain and Cognitive Neuroscience, Liaoning Normal University, Dalian, China; ^2^School of Psychology, Northwest Normal University, Lanzhou, China

**Keywords:** reward learning, emotional face, task irrelevant, attentional capture, distractor

## Abstract

The aim of this study was to explore whether reward learning would affect the processing of targets when an emotional stimulus was task irrelevant. In the current study, using a visual search paradigm to establish an association between emotional faces and reward, an emotional face appeared as a task-irrelevant distractor during the test after reward learning, and participants were asked to judge the orientation of a line on the face. In experiment 1, no significant difference was found between the high reward-fear distractor condition and the no reward-neutral condition, but the response times of the high reward-fear condition were significantly longer than those of the low reward-happy condition. In experiment 2, there was no significant difference in participants’ performance between high reward-happy and no reward-neutral responses. In addition, response times of the low reward-fear condition wear significantly longer than those of the high reward-happy and no reward-neutral conditions. The results show that reward learning affects attention bias of task-irrelevant emotional faces even when reward is absent. Moreover, the high reward selection history is more effective in weakening the emotional advantage of the processing advantage than the low reward.

## Introduction

Attention allocation is affected by reward through modulation of visual salience and behavioral motivation (Boehler et al., 2012; Botvinick and Braver, 2015). In recent years, some researchers have posited that after reward training, even a stimulus as the non-target can still automatically capture one’s attention and thus receive priority processing (Anderson et al., 2011a,b; Awh et al., 2012).

As is known, emotional stimuli always receive priority attention as compared to non-emotional stimuli (Batty and Taylor, 2003; Vimal, 2008; Hodsoll et al., 2011; Barratt and Bundesen, 2012; Ikeda et al., 2013; Schmidt et al., 2015; Pool et al., 2016; Glickman and Lamy, 2017), with the exception of instances with a high percepetion load (Yates et al., 2010; Gupta and Srinivasan, 2015). In addition, early attention bias for negative emotion (such as fearful faces), in which people can quickly detect negative and threatening stimuli, has returned relatively consistent empirical results (Hansen and Hansen, 1988; Luo et al., 2010; Pinkham et al., 2010). This attention preferential processing facilitates us to respond quickly and appropriately to negative stimuli.

When emotional pictures are presented as task irrelevant, the presence of rewards can modulate attention resources and weaken the interference of emotional distractors on target processing (Padmala and Pessoa, 2014; Yokoyama et al., 2015; Walsh et al., 2018). Kulke et al. (2018) asked participants to complete a simple perceptual task while ignoring emotional images. One group was consistently rewarded for completing tasks quickly and accurately, whereas the other group was not rewarded for their performance. The results showed that the presence of rewards could alleviate the disruptive effect of emotions on the processing target (Padmala and Pessoa, 2014; Yokoyama et al., 2015; Padmala et al., 2017). It could be that the presence of rewards enables participants to alter their coping strategies from passive into active control to cope with the changes of the scene and enhance their cognitive control (Botvinick and Braver, 2015).

The association of rewards acquired from past experience can have an important impact on the attention bias. The researchers associated high values with happy faces and low values with angry faces through a reward learning phase, to see whether the reward-stimuli association would affect the processing advantage of threatening faces. The study showed that the preferential processing of anger can be modified by reward learning rather than the impact of endogenous attention during the test (Yao et al., 2013). Reward-modulation effects learned through value association impair early visual perception and hence attention allocation to angry faces. In the later stages of emotional processing, participants employed more cognitive resources to process reward history (Chen and Wei, 2019).

We note that in studies of reward-emotional attention processing, emotional faces appeared as targets during training after the reward is learned, whereas previous studies showed that the combination of reward and goal facilitates target processing (Fan et al., 2014). However, it is still unknown whether reward has an effect on the attention capture of non-target emotional faces when the reward information is absent. In addition, in some studies, reward information (reward cues or feedback) and stimuli were presented at the same trial (Bijleveld et al., 2010; Hickey et al., 2011; Wei et al., 2014, 2015), which inevitably activated reward expectation or reaction motivation of individuals. Unlike the cue paradigm or feedback paradigm, reward learning has a strong shaping effect on individual behavior and mental processing (Libera and Chelazzi, 2014), which can result in the avoidance of the influence of reward expectation and motivation on attention processing (Hammerschmidt et al., 2018). Therefore, our study established an association between emotional faces and rewards through reward learning, and explored whether the reward learning would affect the processing of targets when the emotional stimuli appeared as task-irrelevant distractors and also when reward information is absent during the testing phase. The study consists of two experiments. Experiment 1 was designed to investigate the processing characteristics of low reward-happy and high reward-fearful faces during the test phase after establishing a learning association. Considering the different reward values, their association had different effects on attention selection (Anderson et al., 2011a). Experiment 2 was aimed at investigating the attention processing of high reward-happy and low reward-fear conditions during test phase after reward associations were established. Based on previous studies regarding the relationship between reward and emotional processing, we hypothesized that, after the reward learning, the interference effect of the emotional face would be alleviated in the testing phase when it was presented to be task irrelevant, especially in cases involving a fearful face.

## Experiment 1

### Methods

#### Participants

Twenty-two students (14 female, 8 male; mean age, 20.36 years; age range, 18–24 years) from Liaoning Normal University participated in the experiment. They were all right handed and had either normal or corrected-to-normal vision. The research protocol was approved by the Research Center of Brain and Cognitive Neuroscience, Liaoning Normal University Institutional Review Board, and informed consent forms were signed by all participants. Our rationale for sample size was based on previous studies (e.g., Jahfari and Theeuwes, 2016) and obtained in G-power by setting the partial *η*^2^ as 0.25, *α* as 0.05, and power (1 − *β*) as 0.8.

#### Stimuli

Pictures depicting emotions were chosen from the China Facial Affective Picture System (Huang et al., 2011). Three types of emotion faces were used: happy (*N* = 6; male 3, female 3); fearful (*N* = 6; male 3, female 3); and neutral (*N* = 6; male 3, female 3). To ensure the consistency of material stimuli, we matched the arousal and the facial attractiveness. Twelve participants assessed the valence, arousal, and attractiveness of the emotional faces. There were significant differences in the valence of the three emotions [*F*(2,11) = 141.36, *p* < 0.001, $\eta_{p}^{2}$ = 0.357; happy (2.89 ± 0.45), neutral (4.52 ± 0.32), and fearful (6.32 ± 0.62)]. Happy and fearful faces used here were matched on their arousal [mean (*M*) ± SD, happy (5.33 ± 0.21), fear (5.82 ± 0.35), *t*_12_ = 2.97, *p* = 0.94], the arousal of neutral faces was 3.72 ± 0.32. There was no significant difference in facial attractiveness [*F*(2,11) = 128.68, *p* = 0.69,$\eta_{p}^{2}$ = 0.025; happy (4.23 ± 0.35), neutral (4.12 ± 0.32), fearful (4.08 ± 0.44)]. Luminance was controlled for in the emotional faces using a unified template in Photoshop CS6, and we added line segments in different directions (vertical, horizontal, or oblique) between the eyebrows.

#### Procedure

**Figure 1:**
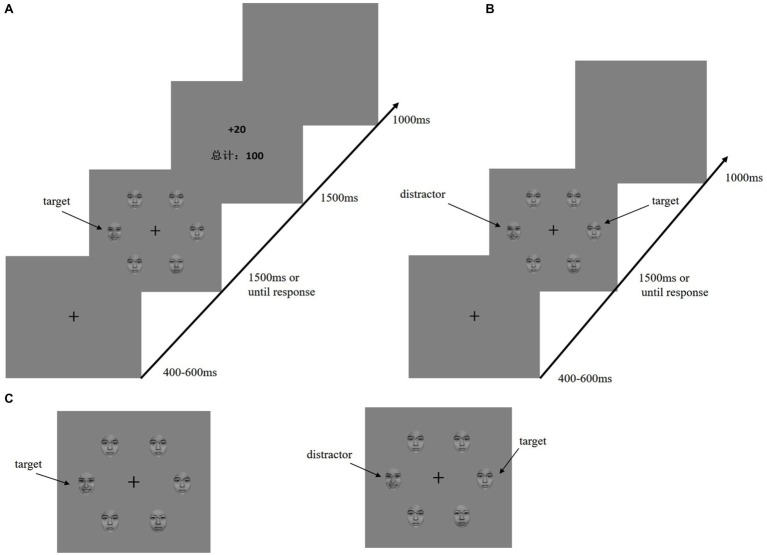
Sequence of trial events. **(A)** Target of training phase was defined by the line of eyebrows on the emotional faces (happy or fearful, only one was present on each trial). Participants must report the line segment inside of the emotion target (horizontal or vertical). Only the correct response will be rewarded. **(B)** Test phase; the target was defined as a neutral-emotion face with a horizontal or vertical line. The distractor was formerly rewarded emotion face. **(C)** To the left is the target of the training phase; to the right is the target and distractor of the testing phase.

In an electromagnetic-shielded room, the participants were seated comfortably approximately 80 cm away from a 17-inch cathode-ray tube screen display. They performed a visual search task adapted from Anderson et al. (2011a,b). The time course for the reward learning and testing phases is shown in Figures 1A,B. During both the training and test phases, the fixation display included a black fixation cross (0.5° × 0.5° visual angle) presented in the center of the display against a gray background, and the search display consisted of the fixation cross surrounded by six emotional faces (3.58° × 3.58° visual angle of each face). The diameter of the emotional stimulation was 10.7°.

##### Training Phase

In the training phase, a baseline block of 120 trials was given to the participants. During the baseline, emotional faces (happy or fearful) were presented as background (there were six faces, one of which was happy or fearful), and one of the neutral faces had a horizontal or vertical line between the eyebrows. The participants were asked to ignore the face and judge the line orientation. The rewarding phase had six learning blocks (540 trials). Six emotional faces (one was a fearful or happy face; the other are neutral faces) were presented in the search display. Furthermore, the target for each trial was a unique emotional face with either a vertical or horizontal black line between the eyebrows Figure 1C. The participants were required to press the F or J key as quickly as possible when judging whether the line orientation between the eyebrows was horizontal or vertical. After a correct reaction by the participants, the corresponding reward feedback and total score appeared on the screen.

Fearful faces (high reward) were followed by “+100 points” feedback at 80% percent, and the remainder was “+20 points.” For happy faces (low reward), the percentages were reversed. It should be pointed out that 500 points were the equivalent of 1 Chinese Yuan Renminbi. Participants were clearly informed that their additional monetary reward was determined by the total points they earned.

##### Test Phase

After finishing the training phase and resting for an hour, all participants completed a second visual search task in which they identified the orientation of a line between the eyebrows. During the test phase, the search display consisted of a fearful face or a happy face among neutral faces, and the target was defined as the horizontal or vertical line between the eyebrows (Figure 1C). The participants were told that the experimental trials would not contain a reward. For the test phase, each trial began with a fixed display (400–600 ms). Next, a search display lasted 1,500 ms until the participant responded. An interval appeared after the response. The test contained no reward feedback, only a blank screen lasting 1,000 ms. The test contained one practice block (12 trials) and six formal blocks (each 160 trials). In the test phase, to ensure that the participants could observe the attention capture effect, 50% of the trials were no-reward distractor stimuli. In the remaining 50% of the trials, 25% were high-reward disturbance stimuli, and the remaining 25% were low-reward distractors.

### Results

#### Training Results

**Figure 2:**
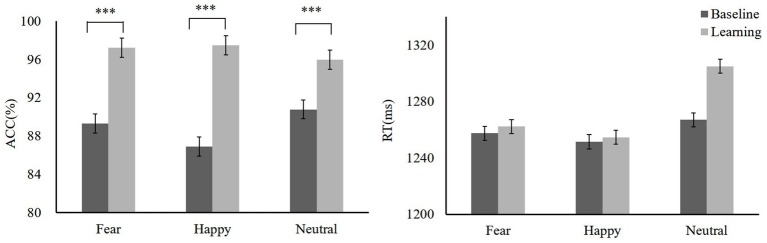
Mean accuracy (ACC) and response times (RTs) of training phase, baseline, and training. Mean ACC to high reward-fear, low reward-happy, and no reward-neutral increased compared with baseline (experiment 1). ***p < 0.001.

During the training, we found that the mean accuracy (ACC) for high reward-fear [*t*(19) = −5.03, *p* < 0.001, Cohen’s *d* = 0.39] and low reward-happy [*t*(19) = −8.19, *p* < 0.001, Cohen’s *d* = 1.72] conditions increased as compared to baseline (baseline: fear 89.31 ± 7.89, happy 86.89 ± 6.39, neutral 90.79 ± 5.93; reward training: high reward-fear 97.25 ± 2.81, low reward-happy 97.49 ± 2.63, neutral 95.98 ± 3.93). A repeated-measures ANOVA showed that ACC differed significantly among these three conditions [*F*(2,38) = 4.04, *p* = 0.025, $\eta_{p}^{2}$= 0.16]. There was no significant difference between high reward-fear and low reward-happy conditions [*t*(19) = −0.79, *p* = 0.44, Cohen’s *d* = 0.03]. In addition, the difference between low reward-happy and neutral did not reach significance [*t*(19) = 1.96, *p* = 0.06, Cohen’s *d* = 0.03]. However, there was a significant difference between low reward-happy and neutral [*t*(19) = 2.21, *p* = 0.04, Cohen’s *d* = 0.31]. The results of the response times (RTs) between reward learning and baseline were not significantly different (baseline: fear 1257.46 ± 78.71 ms, happy 1251.40 ± 88.05 ms, neutral 1266.93 ± 80.73 ms; reward training: high reward-fear, 1262.20 ± 133.1 ms, low reward-happy 1254.57 ± 113.41 ms, neutral 1304.96 ± 105 ms). The training phase data are shown in Figure 2.

#### Test Results

**Figure 3:**
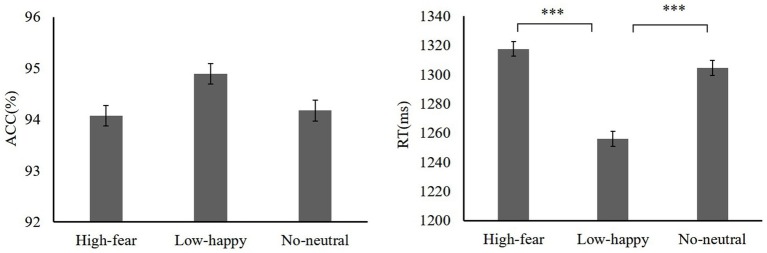
Mean ACC and RTs in different reward distractor conditions (experiment 1). ***p < 0.001.

Next, we examined how reward experiences affected the face section of the test. A repeated-measures ANOVA on accuracy of reward distractor types was used (high reward-fear 94.07 ± 3.23, low reward-happy 94.89 ± 2.34, neutral 94.17 ± 3.52). There was no main effect on distractor [*F*(2,38) = 2.27, *p* = 0.11, $\eta_{p}^{2}$= 0.09]. A repeated-measures ANOVA showed that RTs differed significantly among distractor conditions (high reward-fear 1317.49 ± 87.53 ms, low reward-happy 1256.01 ± 65.83 ms, no distractors 1304.58 ± 77.51 ms) [*F*(2,38) = 10.44, *p* < 0.001, $\eta_{p}^{2}$= 0.54]. A high reward-fear distractor slowed RTs relative to low-happy [*t*(19) = 3.77, *p* = 0.001, Cohen’s *d* = 0.78]. The test phase data are shown in Figure 3.

## Experiment 2

Experiment 1 provided evidence that emotional faces interfered with target processing after reward learning. However, that part of the experiment trained only the low-happy and the high-fear emotions, and not the high-happy and low-fear emotions. In addition, high rewards can give individuals a positive experience, but we wanted to discover whether the combination of high reward-positive emotion generates a stronger attention bias than the negative emotion-low reward. Therefore, in experiment 2, we used the happy-high and fear-low reward combinations in the reward learning phase. The sample size obtained was the same as experiment 1.

### Methods

#### Participants

Twenty students (12 female 8 male; mean age, 21.8 years; age range, 18–25 years) from Liaoning Normal University participated in this experiment. They were all right handed and had either normal or corrected-to-normal vision. The research protocol was approved by the Brain and Cognitive Neuroscience Research Center, Liaoning Normal University Institutional Review Board, and informed consent forms were signed by all participants.

#### Stimulus, Procedure, and Data Analysis

The stimuli, apparatus, procedure, and analysis were identical to those of experiment 1 with the following exceptions. During training, participants were asked to press F or J as quickly as possible whenever they saw a fearful or happy face with either a vertical or horizontal line. Then, a visual feedback informed the participant of the reward earned in that trial, as well as about the total reward accumulated across the trial. Fearful faces low reward were followed by “+20 points” in 80%; the remainder was “+100 points.” For happy faces (high reward), the percentage was reversed.

### Results

#### Training Results

**Figure 4:**
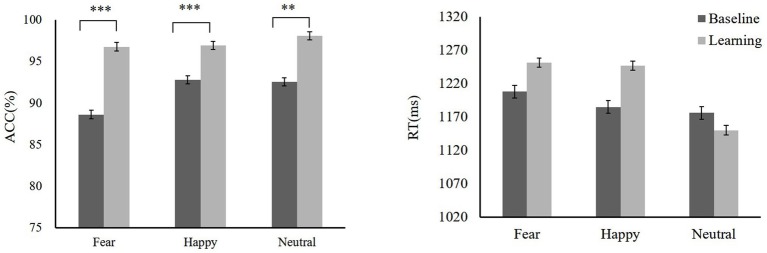
Mean ACC and RTs of training phase, baseline, and training. Mean ACC to low reward-fear, high reward-happy, and no0020reward-neutral increased compared with baseline (experiment 2). **p < 0.01; ***p < 0.001.

During the training, we compared baseline with training. Participants showed high ACC to high reward-happy [*t*(19) = −4.18, *p* = 0.001, Cohen’s *d* = 0.96] and low reward-fear [*t*(19) = −4.61, *p* < 0.001, Cohen’s *d* = 1.06] compared with the baseline (baseline: fear 88.63 ± 3.35, happy 92.79 ± 2.73, neutral 92.58 ± 3.59; reward training: low reward-fear 96.79 ± 2.15, high reward-happy 96.94 ± 4.38, neutral 98.1 ± 3.24). There was no significant difference in RTs between reward training and baseline except in high reward-happy compared with happy [baseline: fear 1207.67 ± 65.25 ms, happy 1184.82 ± 121.8 ms, neutral 1175.96 ± 73.53 ms; reward training: low reward-fear 1251.11 ± 89.92 ms, high reward-happy 1246.49 ± 94.57 ms, neutral 1149.89 ± 114.83 ms; *t*(19) = −2.26, *p* = 0.016, Cohen’s *d* = 0.61]. A repeated-measures ANOVA showed that ACCs among rewards are significantly different [*F*(2,38) = 4.32, *p* = 0.021,$\eta_{p}^{2}$ = 0.19]. Low reward fear ACC is lower than neutral [*t*(19) = −2.5, *p* = 0.022, Cohen’s *d* = 0.58], and high-reward happy is lower than neutral [*t*(19) = −2.09, *p* = 0.05, Cohen’s *d* = 0.48]. There is also a significant difference in RTs for reward types after reward learning [*F*(2,38) = 16.18, *p* < 0.001, $\eta_{p}^{2}$= 0.47]. The training phase data are shown in Figure 4.

#### Test Results

**Figure 5:**
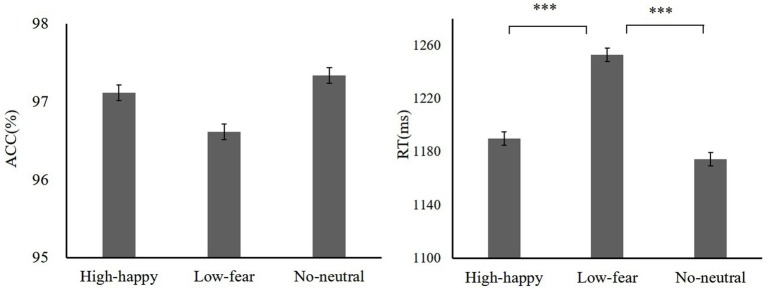
Mean ACC and RTs in different reward distractor conditions (experiment 2). ***p < 0.001.

We wanted to know the impact of previous learning on performance when performance was not predictive of reward. An ANOVA was conducted on the ACC with reward condition. The results showed that there was no difference of reward [*F*(2,38) = 1.07, *p* = 0.19,$\eta_{p}^{2}$ = 0.04; high reward-happy 97.11 ± 7.54, low reward-fear 96.61 ± 5.47, neutral 97.33 ± 3.41]. The analysis of ANOVA on the RT indicated that there are significant differences between reward types [*F*(2,38) = 18.76, *p* < 0.001,$\eta_{p}^{2}$ = 0.14; high reward-happy 1189.84 ± 118.12 ms, low reward-fear 1253.07 ± 120.40 ms, no distractors 1174.35 ± 97.67 ms]. Paired comparisons showed significant differences between high reward-happy and low reward-fear (*p* = 0.001). There was a significant difference between low reward-fear and no reward-neutral (*p* = 0.001), but there was no difference between high reward-happy and no reward-neutral. The test phase data are shown in Figure 5.

### Between-Experiments Comparison

The ACC data were evaluated with a 2(Reward: high and low) × 2(Emotion: fearful and happy) repeated-measures ANOVA. The main effects of reward (*F* < 1) and emotion (*F* < 1) were not significant, and the interaction of reward × emotion was also not significant [*F* = 2.87, *p* = 0.109,$\eta_{p}^{2}$= 0.14].

**Figure 6:**
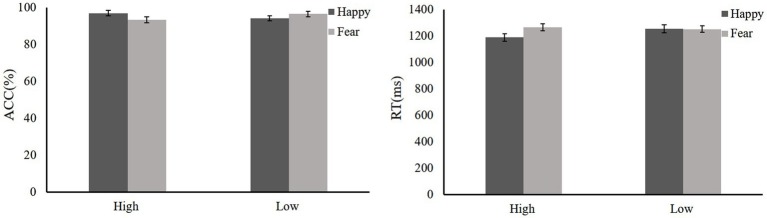
Mean ACC and RTs in different reward distractor and emotions.

We also evaluated the RT data according to a 2(Reward: high and low) × 2(Emotion: fear and happy) repeated-measures ANOVA. The main effect of reward was not significant (*F* < 1), and the mean RTs of high reward and low reward were the same. The main effect of the emotion was significant [*F*(1,36) = 28.9, *p <* 0.001], and the mean RT of the fearful face distractor was slower than that for the happy face. Critically, a significant Reward × Emotion interaction was detected [*F*(1,36) = 5.31, *p* = 0.034,$\eta_{p}^{2}$ = 0.24]. The results of simple effect analysis showed that there was a significant difference in the fearful face distractor between high and low reward conditions [*F*(1,36) = 5.73, *p* = 0.029]. High reward is slower than low reward. Moreover, there were significant differences in emotion faces [*F*(1,36) = 14.61, *p* = 0.001]. The fearful distractors (1317.49 ms) were longer than the happy distractors (1189.84 ms) in the high-reward condition; there was no such significant difference in the low-reward condition (*F* < 1). The group comparison data are shown in Figure 6.

## Discussion

In the current study, we used the reward learning paradigm to investigate the effects of reward learning on attention capture of non-target emotional faces. During the training phase, the fearful and happy faces were connected with different rewards (high vs. low rewards). The participants then completed visual search tasks in the testing phase without any reward feedback, in which emotional faces trained in reward learning were presented as non-target distractors. The results showed that after the reward training, the ACC of the emotional faces was significantly improved compared with the baseline. During the test phase, no significant difference was found between high reward-fear and no reward-neutral conditions, or between high reward-happy and no reward-neutral conditions. The RTs were shorter in the low reward-happy condition than in the high reward-fear and no reward-neutral conditions. In addition, the RT was longer for low reward-fear versus high reward-happy and no reward-neutral conditions. The results suggest that the reward selection history perhaps changed the attentional selection of non-target emotional faces after high-reward learning.

Reward has a strong effect on cognition and can allocate a mass of cognitive resources to reward-related stimuli (Wei et al., 2014). During reward training, fearful and happy faces were presented as the target background, and the attention resources of the participants could be biased toward the facial background (Langton et al., 2008; Wentura et al., 2011). During such training, participants may have implicitly learned an association between the emotional faces and the rewards. Reward feedback enhances the response motivation and improves the performance of target processing (Anderson et al., 2011b; Anderson, 2016).

In the test of experiment 1, no difference was confirmed between the high reward-fear distractor condition and the no reward-neutral condition, but the RTs of the high reward-fear condition were longer than those of the low reward-happy condition. It is well known that threatening stimuli (e.g., fear, anger) could capture the individuals’ attention, despite the fact that they were irrelevant to the current goal (Batty and Taylor, 2003; Barratt and Bundesen, 2012; Ikeda et al., 2013; Bucker et al., 2014). However, the current results suggest that processing a fearful face does not generate attention disengagement difficulties. Recent studies regarding reward and emotional processing found that the processing advantage of irrelevant negative stimuli was impaired under reward conditions (Schettino et al., 2013; Padmala and Pessoa, 2014; Yokoyama et al., 2015). In Padmala and Pessoa’s (2014) study, however, rewards and tasks were presented in the same stimulus sequence. In our study, reward feedback did not appear during the test. Results still show that the value association between high reward and fearful faces still weakens the processing advantage of fearful faces when the reward feedback is absent. Because of the high reward-fear association acquired, the participants may have adopted an active strategy, which weakens the distraction of irrelevant stimuli on the target searching. It further showed that reward training can effectively regulate the processing of non-target fearful faces, and that the reward-learning effect persisted even when the reward does not appear.

In the test of experiment 2, there was no difference in participants’ performance between high reward-happy and no reward-neutral conditions. In addition, the RTs of the low reward-fear condition were longer than those of the high reward-happy and no reward-neutral. It is rather easy to produce perceptual priming with positive emotions in social life situations, which is very common and familiar (Öhman et al., 2001). After the learning between happy face and high reward, it may enhance positive emotional experience, causing the participant to react faster to the happy face distractor. Unlike previous studies, the fearful face was not affected by low-reward learning and still showed a negative processing bias (Itthipuripat et al., 2015; Yokoyama et al., 2015; Bourgeois et al., 2017). We supposed that this may be due to the different task paradigms used and task-irrelevant stimulate previously associated with a small reward shows weaker impact than that previously associated with a larger reward (Anderson et al., 2011a). Moreover, in the study by Yokoyama et al. (2015), tasks were relatively easy with less interference. In the current study, the target search is more difficult because of a lot of task-irrelevant stimulate.

Previous studies concerning reward learning showed that different rewards have disparate effects on attention processing after reward learning. More specifically, task-irrelevant distractor previously associated with a large reward slows visual search more than an equally salient distractor previously associated with a small reward (Anderson et al., 2011a,b; Anderson, 2013). Compared with low reward, a high-reward selection experience could alleviate the disturbance effect introduced by a non-target emotional face and promote the target recognition; this finding seems to be inconsistent with the results of previous studies (Padmala and Pessoa, 2014; Yokoyama et al., 2015). On the whole, on the one hand, individuals may reallocate more cognitive resources to evaluate high-reward selection history during the decision-making stage of the emotional processing (Chen and Wei, 2019). On the other hand, the difference between the participants in experiment 1 and experiment 2 may also lead to this result. The reward learning weakens the processing advantage of the fearful face. However, the high reward-happy faces were less likely to interfere with the goal, probably because positive emotion is more common in real life.

Previous research regarding this issue usually presented rewards and goal tasks sequentially. Such a paradigm setting would increase the motivation of participants. The present study associated rewards with emotional faces in an independent training phase. During the testing, reward-associated stimuli appeared to be task irrelevant, and the reward effect can be observed indirectly. In addition, the current study used the China Facial Affective Picture System to study the emotion processing in a Chinese cultural context. The limitation is that this study chose only two types of emotional faces (happy and fearful). Thus, the discussion of how rewards influence emotional processing is simplified. Owing to the complex nature of emotional stimuli and the difficulty of the task, the reward-learning effect may be weakened. Future research will explore the impact of reward learning on other emotions, thereby appropriately reducing the difficulty of the task.

## Conclusion

The current study investigated the impact of reward learning on emotional attentional capture and provided evidence for relationships between reward learning and emotional faces. The results showed that RTs for high reward-emotional faces distracters are faster than those for low reward-emotional faces. Furthermore, no significant difference was confirmed between the high reward-fear distractor condition and the no reward-neutral condition. We speculate that reward learning affects the attention bias of task-irrelevant emotional faces even when the reward is absent. Furthermore, reward selection history influences the attention bias of emotional faces, in which emotional faces are connected to high reward. Specifically, the attention advantages of fearful faces were regulated by high reward.

## Data Availability Statement

The datasets generated for this study are available on request to the corresponding author.

## Ethics Statement

This study was carried out in accordance with the recommendations of “Brain and Cognitive Neuroscience Research Centre, Liaoning Normal University Institutional Review Board” with written informed consent from all subjects. All subjects gave written informed consent in accordance with the Declaration of Helsinki. The protocol was approved by the “Liaoning Normal University Institutional Review Board.”

## Author Contributions

XZ and ZW designed the experiment. BD collected the data. XZ analyzed relevant data and wrote the manuscript. WH revised the paper and contributed to the interpretation of the data for the work.

### Conflict of Interest

The authors declare that the research was conducted in the absence of any commercial or financial relationships that could be construed as a potential conflict of interest.
